# Violence and reproductive health preceding flight from war: accounts from Somali born women in Sweden

**DOI:** 10.1186/1471-2458-14-892

**Published:** 2014-08-30

**Authors:** Ulrika Byrskog, Pia Olsson, Birgitta Essén, Marie Klingberg Allvin

**Affiliations:** School of Education, Health and Social Studies, Dalarna University, S-791 88 Falun, Sweden; Department of Women’s and Children’s Health, Uppsala University, Akademiska sjukhuset, S-751 85 Uppsala, Sweden; Centre for Clinical Research, Nissers väg 3, S-791 82 Falun, Sweden

**Keywords:** Somalia, War, Violence, Refugee, Sexual and reproductive health and rights, Qualitative method, Thematic analysis

## Abstract

**Background:**

Political violence and war are push factors for migration and social determinants of health among migrants. Somali migration to Sweden has increased threefold since 2004, and now comprises refugees with more than 20 years of war experiences. Health is influenced by earlier life experiences with adverse sexual and reproductive health, violence, and mental distress being linked. Adverse pregnancy outcomes are reported among Somali born refugees in high-income countries. The aim of this study was to explore experiences and perceptions on war, violence, and reproductive health before migration among Somali born women in Sweden.

**Method:**

Qualitative semi-structured individual interviews were conducted with 17 Somali born refugee women of fertile age living in Sweden. Thematic analysis was applied.

**Results:**

Before migration, widespread war-related violence in the community had created fear, separation, and interruption in daily life in Somalia, and power based restrictions limited access to reproductive health services. The lack of justice and support for women exposed to non-partner sexual violence or intimate partner violence reinforced the risk of shame, stigmatization, and silence. Social networks, stoicism, and faith constituted survival strategies in the context of war.

**Conclusions:**

Several factors reinforced non-disclosure of violence exposure among the Somali born women before migration. Therefore, violence-related illness might be overlooked in the health care system. Survival strategies shaped by war contain resources for resilience and enhancement of well-being and sexual and reproductive health and rights in receiving countries after migration.

## Background

Violence targets women worldwide and female refugees are at increased risk for of violence exposure due to conflict, instability, and displacement [1, 2]. Within maternity health care systems, refugee women need targeted attention, as violence is associated with suboptimal sexual and reproductive health (SRH) [3, 4]. In order to offer adequate health care and support, health care providers need awareness of context specific features and diversities of patients’ previous experiences [5].

The collapse of the Somali state in 1991 left the country in civil war and with a ruined infrastructure [6]. Repeated refugee movements have followed. Since 2006, approximately 760 000 Somalis are estimated to have left their country due to war and drought [7]. The first large group of Somali refugees arrived in Sweden in the beginning of the 1990s. The second larger group has arrived since 2007 [8], and comprises refugees with more than 20 years of war experience. At the end of 2013, about 45 000 Somali born individuals were living in Sweden [8].

In 2008, the maternal mortality rate in Somalia was estimated at 1200 deaths per 100 000 live births, which ranks the second highest in the world [6]. At the end of the 1990s, perinatal mortality after migration, among Somali refugees in high-income countries, including Sweden, was high [9–12]. Remaining suboptimal outcomes and higher levels of maternal ill health during pregnancy, compared to Swedish born women [13, 14], and late access to maternity health care services are reported [14]. The strategies Somali born women has adopted as a result of pre-migration experiences have been suggested to contribute to adverse pregnancy outcomes during the encounter with a western bio-medical maternity health care system [5, 15].

For women and girls, war and migration often reinforce the discrimination already present in different levels of society [2] and in addition there is an increased risk of degraded physical safety and exposure to collective and interpersonal violence in sending and receiving countries and during flight [1, 2, 16]. Past traumatic events, exposure to violence and reproductive ill health are linked [4, 17] and maternal exposure to violence affects perinatal health [4, 18].

Violence is described as ‘avoidable insults to basic human needs’ [19] and is multifaceted in the context of war. WHO recognizes neglect, emotional, physical and sexual violence at self-directed, inter-personal, and collective levels [20], and in accordance with this, the focus of the present study was the social, political, and economical aspects of collective violence, non-partner sexual violence, and intimate partner violence (IPV). The integrated ecological framework [20, 21] was used to understand the interacting factors contributing to violence perpetration, exposure, and the consequences. This framework operates in and between four systems. The individual system includes biological features, personal history and traits. The relational system includes the immediate context and close relationships. The community system explores institutions where social relationships take place, for example schools and neighborhoods, and the societal system includes political and economic structures in society and cultural values [20].

Health is influenced by earlier life experiences; therefore, migrant health must be considered in a life course perspective, both in sending and receiving countries [22], as adverse sexual and reproductive health (SRH), violence, and mental distress are linked [4, 17]. In maternity health care services, it is increasingly common to ask patients about exposure to violence. Antenatal midwives consider immigrants as a vulnerable group at increased risk of violence [23] but have difficulty in addressing their needs [24]. Therefore, exploring Somali born women’s pre-migration experiences of reproductive health and different forms of violence is important for understanding the adverse SRH outcomes among Somali refugee women.

The present paper forms part of a larger data collection process covering aspects before, during, and after migration from Somalia. The aim of this study was to explore experiences and perceptions on war, violence, and reproductive health before migration among Somali born women in Sweden.

## Methods

A qualitative emergent design [25] with individual semi structured interviews and thematic analysis [26] was applied to capture depth and nuances from the participants’ perspectives.

The data collection and reporting adheres to the RATS guidelines for reporting qualitative studies [27].

### Informants

The data collection was performed between December 2011 and December 2012 in mid-Sweden, where the number of Somali refugees has increased fivefold since 2006. Informants were recruited through maternal and child health (MCH) clinics and local Somali networks. Inclusion criteria were 1) Somali born woman 2) in fertile age. After oral and written information about the study all informants gave their oral and written consent for participation. Purposive sampling was applied as we aimed to gain diverse opinions and perspectives of the topic. Thus, informants with a variety of backgrounds based on age, parity, education and years of residence in Sweden were included. The recruitment of informants stopped when little new knowledge was obtained during conducted interviews.

All informants proved to have permanent residence permits in Sweden and their family situations were generally complex. Most informants originated in south-central Somalia. Five informants had left biological children behind during flight and three guardians to siblings or young close relatives had been forced to leave them behind. Ten of the married women were involuntarily separated from their husbands, who were mainly scattered in African countries. Three informants were divorced whereof two had remarried in Sweden. Three informants were pregnant with husbands living abroad at the time for the first interview. Three had experienced the death of own children in Somalia and one informant had experienced a stillbirth in Sweden. Background characteristics are presented in Table 1.

**Table 1:** **Characteristics of informants at first interview (n = 17)**

Age		Current occupation	
18-24	6	Language studies	6
25-34	8	Parental leave	7
35-45	3	Preparation program	1
Education (years)		Paid employment	3
0 or Quran-school*	5	Parity	
Primary school	6	0	3
Middle/Secondary school	1	1	4
High school	4	2-3	8
University	1	4-7	0
Residence Sweden (years)		>7	2
0-1	2	Pregnant	5
1-2	6	Marital status	
2-3	6	Single	2
4-10	1	Cohabiting	2
10-20	2	Married	13
		whereof co-habiting	4
		not cohabiting	9

*Includes recitation, reading and writing Quran-Arabic.

### Interviews

Qualitative semi-structured individual interviews (22) were conducted with 17 Somali born women in mid Sweden. Data collection was carried out in two steps. In the first step, twelve informants were asked to freely narrate about their lives before, during and after migration from Somalia to Sweden. A pilot tested thematic topic guide covered family relations, decision-making, childbearing, violence, war, and strategies enabling survival and well-being during this period. The informants spoke freely but rather superficially on sensitive aspects of migration, violence, and relationships which were of particular interest in this study. To further clarify this focus in the forthcoming interviews we developed a vignette [28] covering these aspects. The vignette was based on information from the interviews in the first step and depicted personal experiences of a fictive Somali born refugee woman’s (Asma) migration to Sweden. The vignette was pilot tested with two Somali interpreters and thereafter shortened.

#### Vignette describing a Somali woman’s experiences of migration to Sweden

*This is the story of Asma. She is not a real person and the story is not true. Asma is brought up in Somalia. She is married and has four children with her husband Mohammed. The situation in Somalia is difficult due to the war and the family decides that Asma has to escape. The three oldest children remain with their father in Somalia. Together with the youngest daughter, she first travels by land, crossing the border to Ethiopia and then by air to Sweden. She is granted a resident permit after some months. She is worried about her children still staying in Somalia and sometimes she cannot sleep during the night. Mohammed has to hide periodically and then the children are scattered with relatives. After two years, Mohammed and the children are allowed to come to Sweden based on family ties. Now they all live together in an apartment, Asma becomes pregnant and looks forward to having another baby. She starts to visit the antenatal care (ANC) clinic. She has friends that she visits regularly. She has also a preparation position for employment after finishing her Swedish studies. In Somalia, Mohammed was employed outside the family, he is not used to her earning money, and he asks her to let him keep the money so she can ask him for money when needed. He is not secure with her being away from home that much and wants her to stay at home more, to care more for the home, the children and him and tells her to ask him if she is allowed to leave the home or not. Sometimes he forces her to stay at home. When Asma suggests they can share the housework, as she is working and he has still not started his language studies, he becomes angry and cannot avoid hitting her. Asma is very uncomfortable being near him and starts to stay away from him more and more, even though he wants her to sleep with him every night. She is also worried that Mohammed will hit the children. At the ANC clinic, the midwife tells her that she should ask all patients she meets whether they have experienced violence at any time. Today, she is asking Asma.*

In the second step, the interviews (10) were initiated by reading this vignette aloud and the informants were asked to reflect upon Asma’s experiences. Probing was used to deepen the informants’ reflections based on a modified topic guide. The vignette-based interviews provided richer information on experiences and perceptions on migration, violence and relationships both pre and post migration. Five of the 10 interviews in the second step were recruited as in the first step. The remaining 5 were follow-up interviews with pregnant women in the first step that had agreed to a follow up interview after the post partum period.

To ensure safety and comfort, interviews were held in privacy in an undisturbed place known to the informants. All interviews were conducted by the first author with assistance of one of two professional Somali interpreters with health care professions. Three interviews were held in Swedish without an interpreter. The interviews were between 50 and 90 minutes long and were digitally recorded with the informants’ permission. After the interviews, the first author and the interpreter reflected on the content, nuances, and interaction during the interview. Field-notes on non-verbal communication and reflections supported reflexivity throughout the research process.

### Data handling and analysis

Data consisted of 26 hours of interviews and all recordings were transcribed verbatim. To verify the accuracy of translations, parts of five interviews in Somali were translated by independent interpreters and compared with the translations to Swedish and minor corrections were made. Inductive thematic analysis was conducted to organize and describe the patterns in data [26]. To gain an overall understanding of the different ways that violence and reproductive health were addressed, the first author listened to all digitally recorded data and read the transcripts. The authors agreed to focus on pre-migration aspects and to report data covering the migration and settlement phase separately in order to enable thorough presentations of the rich data. Thus, first data extracts directly or indirectly describing aspects of violence and reproductive health before migration were identified. From there, numerous codes based on segments of raw data related to the aim of the study were created. The codes were grouped into subthemes and themes, which constitute “…patterned response(s) or meaning(s) within the data set” [26], p 82]. During the process of sorting codes into themes and sub-themes, attention was paid to both recurrent and contrasting patterns in the data. Themes and subthemes were reflected on and revised by the co-authors in a movement between more descriptive and interpretive levels [26]. Repeated comparisons between evolving themes and the original transcripts ensured their relevancy.

### Ethical considerations

As women’s involvement in violence related research may have negative implications if publicly known, an overall neutral public title of the study was used. All informants were informed orally and in writing of the purpose, confidentiality and, procedures of the study, alongside the voluntary nature of their participation. Furthermore, all informants were informed about who to contact if questions or emotional problems arose afterwards. Ethical approval was obtained from the Regional Ethical Review Board of Uppsala, Sweden (2008/226).

## Results

Most informants declared their own exposure to violence or the overall situation of war related violence as reasons for fleeing Somalia. The analysis resulted in three themes and seven sub themes on war, violence, and reproductive health in Somalia (Table 2).

**Table 2:** **Themes and sub**-**themes on war, violence and SRH in Somalia**

Theme	Subthemes
Violence is everywhere in war-torn Somalia	*Controlled by the fear of violence*
	*Interrupted life and scattered families*
	*Childbearing – natural but hazardous*
The silence of sexual and intimate partner violence	*Rape happens, but who can intervene?*
	*Intimate partner violence is a family issue*
Stoic women keep life together	*We cannot dwell on what cannot be changed*
	*We have learnt to be strong*

### Violence is everywhere in war-torn Somalia

Violence as a widespread process that created fear and affected the daily life of families throughout Somalia was a dominant theme. Despite two decades of war, life and childhood in the homeland was mainly described positively - if it had not been for the violence.

#### Controlled by the fear of violence

Violence had permeated the Somali community at all levels. Sudden violence at market places, roads, schoolyards, on buses, and in residential areas was described. Perpetrators were mainly spoken of as national and international soldiers and militia groups. Clan-based violence was mostly connected to the earlier stages of the civil war. Furthermore, a spread of violence among single civil actors was depicted, due to lack of laws, posttraumatic stress, long-term instability, and the use of khat and other drugs. A variety of witnessed or experienced events of violence were described; bombing, shooting, whipping, beating, sexual harassment, rape, threats, and murder.

*“The worst thing I experienced was when someone was killed in front of my eyes. I tried to - I was terrified. I tried to stop the armed perpetrator. ‘In the name of Allah – don’t kill’ I told him. Some people passing by removed me from the man. He threatened me to death.” (Woman 9)*

Face-to-face threats or by telephone were described as militia strategies.

*…they use to behead people. I couldn’t sleep due to fear.[…] Since they used to call me I didn’t sleep. I thought they would slaughter me. They are among the people and it’s not possible to recognize them.” (Woman 6)*

A more pronounced separation between women and men was imposed in local areas, depending on religious interpretations of current rulers. Women’s living space regarding clothing, occupation, and behavior were restricted in parallel with escalated violence. Refusing to wear niqab [a cloth covering the face] in public places could result in being beaten, and continuing a public business could yield imprisonment. Women were the main targets for restrictions but men’s lives were also threatened. Men could be killed for not adhering to local rules, such as prohibiting women other than their own wife to be seated in the front seat of the car. This happened to the husband of one of the informants when he entered another district with a sick woman in his taxi.

*“‘We are going to arrest you, so step down’ they* [the armed men] *said to him. […] Then he said:’I am not going to step down’ […].The man I was married to, he died, he was murdered.” (Woman 4)*

#### Interrupted lives and scattered families

Violence interrupted daily life and caused instability and separation. The reasons behind movements were on-going fights, personal threats, or local rules in different areas. As families frequently had to search for safer space, lives were lived on a day-to-day basis. Multiple interruptions over the years contributed to a low educational level and frustration at not being able to provide for the children’s future.

*“I mean gunshot that doesn’t know whom to kill or not to kill, lack of education, children without education, reduction of education due to shootings”. (Woman 1)*

Livelihood opportunities were disturbed due to the decreased possibilities of keeping work positions or businesses. Marriage and family building could be postponed as some males were forced to hide, or were captured or killed. The traditional right for the bride and her family to ask for financial guarantees before marriage was restricted by militia, which left the woman with less decision power. Moreover, women living in female-headed households could be forced to marry someone chosen by the local armed rulers or to suffer severe penalties.

*“Then they said to me: ‘What do you think about marrying one of these men’? I said I couldn’t marry: ‘I have a small girl’. When I said so, they told me; ‘You have to make up your mind: either you leave within three days or you take* [one of the men]’.” (*Woman 4)*

One response to violence was the emerging phenomena of “buufis” in the community. Informants defined”buufis” as *“when you dislike where you are and want to go to other countries”,* which was described as widespread and affected especially young people. Symptoms, varying from mild to severe, were defined as a circle of an excited emotional state, worry, and depression. “Buufis” occupied the person’s mind to the extent that nothing mattered except leaving the situation in Somalia and this was the only known cure. Informants shared their own experiences or stories of relatives who had lived in this state of limbo for long periods, which caused major concerns within families and mobilizing them towards deciding to support migration.

The decision to migrate was mostly a joint family agreement. The primary focus was the future of their children and the wellbeing of the larger family. Individual freedom, own will, and the longing for peace were other reasons mentioned, especially when lives were at risk. Social networks, mainly the extended family were described as playing crucial roles in arranging flights and engaging relatives still in Somalia or overseas.

*“When I told my brother* [about the threat of coercive marriage] *he said: ‘Leave, I will send someone for you. Before you lose your life’.* […] *It was only me that left. The little girl* [small sister] *is still there.” (Woman 4)*

#### Childbearing –natural but hazardous

Motherhood and childbearing were delineated as strong identity markers for women. However, escalating violence targeted this core value by separating couples and limiting possibilities for early bonding between mother and child. Despite the barriers and insecurity, motherhood was maintained and family building, conceiving, and childbirth continued as far as it was possible. Feelings, hesitations, or strategies questioning childbearing in a country dominated by violence and war were rarely expressed by informants.

Limited access to qualified health care and intense fear during birth due to ongoing warfare were described. However, the difficulties of childbearing such as pain, lack of information or support, fear of death, and feelings of insecurity were also normalized. For some informants, these features were associated with the natural life cycle of a woman and something every woman passes through with strength.

*“…the desire* [to have children] *is stronger than the pain. If I want something I would struggle for it, even if it is difficult and pain and hard. […] It is good to have children, it is good to become a mother. It is good both for the life and later. This is why I would struggle to get children”. (Woman 4)*

Regular check-ups during pregnancy were not available but doctors were sometimes consulted when a woman encountered a health problem during pregnancy. Social networks were crucial for support connected to childbearing and some informants hired private doctors, midwives, or traditional birth attendees to assist them during home births. In case of serious illness connected to childbirth, the family could be mobilized to arrange transportation and care in a neighboring country.

Increased reproductive health risks affected women and children during the later years of warfare. In certain areas, depending on the current local rulers, male doctors were not allowed to care for women during birth, which decreased the already limited access to skilled birth attendants. Receiving help from a male doctor at home could result in physical assault and death.

*“…they* [the militia men] *abused me. […] They think that if someone happens to see the genital organs of a married woman, the woman has to be stoned – stoned to death. […] It was just after the delivery I escaped. I had recently given birth.” (Woman 5)*

To escape violence, a last resort was to entrust the care of a newborn baby to other family members. This left mothers with feelings of sorrow over interrupted breastfeeding and shortcomings of not being there for their children.

*“I swear, my feeling was very difficult…She was 17 days old when I left my baby. I brought two children older than her. The circumstance did not allow me to take her with me.” (Woman 1)*

### The silence of sexual and intimate partner violence

The second theme comprised non-partner sexual violence and intimate partner violence (IPV) as a known but silent phenomenon. These issues were only broached during the interviews after direct questions and the informants did not reveal own experiences but shared stories in the third person.

#### Rape happens, but who can intervene?

Fear of non-partner rape and sexual harassment restricted the women’s daily life and travelling and moving around after darkness was particularly hazardous for women. However, sexual violence was not restricted to those occasions. Both civilians and different fractions of soldiers in and close to Somalia were described as perpetrators and the threat of sexual violence or coercive marriages were triggering factors for finalizing flight.

In the community, attitudes towards rape were characterized by ambiguity. Despite awareness about forced sexual actions, the woman’s possibly voluntariness in the act was also a question for gossip. Thus, gossip and the subsequent risk of stigmatization and isolation restricted a woman’s disclosure of rape, because she would have to carry the shame.

*”It is shameful to us if a person gets raped. Wrong things can happen. For the person, it is shameful to tell. The raped person believes that she should not be among people.” (Woman 5)*

Being raped was considered even more devastating if it was a ‘young woman’, a virgin. A “used” woman would have difficulties in getting married, and if a woman refrained from taking action after violence exposure, it would be easier for her and her family to have an ordinary life and escape the shame. One informant reflected over the sadness of keeping sexual violence a secret; if it had happened in Sweden to a Swedish girl, the mother would have dared to take action, to comfort and provide societal help for her daughter, whereas, back in Somalia, a girl would have to bear it by herself.

The escalations of sexual violence were parallel to the war-related breakdown of health care. The existing, although limited, health care system was portrayed as only handling physical needs. This, combined with limited knowledge on organized psychological help also contributed to the silence in the aftermath of rape.

*“This with rape, I think is something new in Somalia. So, the general public or the people have not learned how to support and what kind of help is available. There are different women’s organizations helping. Not many know they exist. And before the war I don’t think there has been such an organization helping women that have been exposed to violence […] So, I think it will be that you keep it to yourself, in the small family.” (Woman 17)*

The lack of laws and regulation had undermined women’s right to receive juridical support.

*“…but who can intervene? There is no government that can intervene – in such a situation you are forced to keep quiet or forget it.” (Woman 11)*

Sometimes families would take the law into their own hands and punish the perpetrator. If the woman was unmarried, one option could be to move to another part of the country or abroad or to arrange a marriage within the extended family. Arranging a marriage with the perpetrator was described as an old traditional way, which was rare but still used. The honor of the family was mentioned but also a will of support and protection towards the girl or woman.

*“…Now this with honor related violence…No one in the family would even think of killing a woman. They would save the own daughter. And then the whole family would help each other and she would marry one of the cousins.” (Woman 17)*

Pregnancy due to rape brought complications for women, children, and families. Induced abortions were described as rare, forbidden, and not accepted within society according to Islamic faith. Relational problems between husband and wife was mentioned. The best way for a married woman was to pretend that the child had the same father as her other children. A child born outside marriage would suffer, as it would be difficult for the family to relate to the child, they were described as traditionally treated badly in families and by the public. Another worry was identity problems for the child when the father was unknown.

*“It might be that the reasonable husband shows tolerance, but the child will not be left in peace – not even in adult ages. The child will discover that the person is not the biological father. People in the community will talk about it. They will say: ‘You’re a bastard – You’re a bastard’.”(Woman 13)*

#### Intimate partner violence is a family issue

The phenomenon of IPV was described as over shadowed by war and war-related violence within the community, and the informants had diverging views on the occurrence and risk of IPV.

Some informants had seldom heard of IPV in Somalia, and considered it a predominantly rural phenomenon, particularly among the nomads. A traditional saying conveyed a respectful attitude towards married women. Marriage was depicted as a rope between a woman and a man; you only own the rope, not the woman at the other end. Furthermore, IPV was portrayed as abnormal and forbidden in Islam.

*“…To me it* [wife-beating] *is nearly uncommon […] in Islam, it’s not included. And if you are religious, you know you shall not do it.” (Woman 15)*

If actions of IPV occurred, it was considered an issue to be solved within the marriage and within the larger family. Other informants considered that the widespread perception of IPV as an internal issue contributed to a high risk and prevalence of IPV with the lack of a juridical system reinforcing this risk. In their view, women were characterized as being without rights and at the mercy of husbands and family decisions.

*“Even if he does something wrong, people say ‘It’s his wife, let him do as he wants’.” (Woman 5)*

However, there were occasions when women who were not family members intervened in marital issues.

*“Then he* [a newlywed husband] *came outside, took a big stone, put it on her* [the young wife’s] *back and tried to rape her, while we were able to watch. So we, the girls, ran to him, removed him and then hid her for two days. Then we sent her back to the village she came from.” (Woman 5)*

### Stoic women keep life together

#### We cannot dwell on what cannot be changed

For the informants, the overall situation of war in the country was impossible to alter and many instances of loss and violence just had to be accepted.

*“You go away to provide for your children and when you come back all can be gone by a grenade. Your children, your home, everything and there is nothing you can do about it.” (Woman 1)*

Life went on and people had to adjust to the circumstances. Paying too much attention to own fears or doubts would be useless, even though feelings of anxiety were connected to life in Somalia, the flight, and insecure future. Strong enabling factors in life were toleration, and having patience and strength in life without ruminating on the past or the future. Hence, the informants’ responses to violence were characterized by pragmatism. Violence had shaped the way of expressing feelings and the way of living and acting. Living in and leaving a war torn homeland was painful and had a devastating impact on life, but a pragmatic view on life made survival possible.

*“…You try to forget. You cannot change things that have happened in the past, even if you think of it the whole time or cry about it.” (Woman 9)*

#### We have learnt to be strong

Women’s strength, decisiveness, and capability at community, relational, and individual levels were prominent in the interviews. These characteristics were described as central to keeping their own and family life together and to overcoming demanding daily obstacles. Women were described as increasingly active in family decisions and public life, running small businesses, and supporting each other in childcare. Additionally, they adjusted to political changes by moving to safer areas and in the search for livelihood. The women kept contact with relatives in Somalia and abroad and prepared for flight. Their ability to be strong and move forward was sharpened by the long-lasting war.

*“We got strength from all the difficulties we have been through during the war. Everything we went through gave us strength and made us heroic.” (Women 6)*

A foundation for the expressed capability was found in an upbringing where strength and ability to manage situations had been emphasized. The deeply rooted Islamic belief was referred to as a source of both strength and future-orientation on the individual level.

*“You shouldn’t just say, I can’t manage, I can’t manage this, you shouldn’t say. When we grew up we didn’t hear those words ‘You never manage this’. I have never heard this from my parents, other parents, the Quran-school […] so, the culture gives very much, that we look forward only, that I can make this…” (Woman 15)*

## Discussion

The present interview study with Somali refugee women in Sweden explored their views and experiences of violence and SRH in Somalia. Our findings revealed escalating violence within the community with subsequent risks of direct violence exposure and violated sexual and reproductive health and rights. Although women were described as capable when handling the consequences of war-related violence, few options were available for women exposed to non-partner sexual violence or IPV.

### Silenced violence and violated sexual and reproductive health rights

Narratives of coercive marriages, sexual violence, IPV, and restrictions limiting sexual and reproductive health care highlighted various forms of gender-based violence and violations of sexual and reproductive health and rights (SRHR). Few informants revealed self-experienced non-partner sexual violence or IPV but described actions of sexual violence in the surroundings as triggering factors for them to escape and avoid the risk of being exposed.

**Figure 1:**
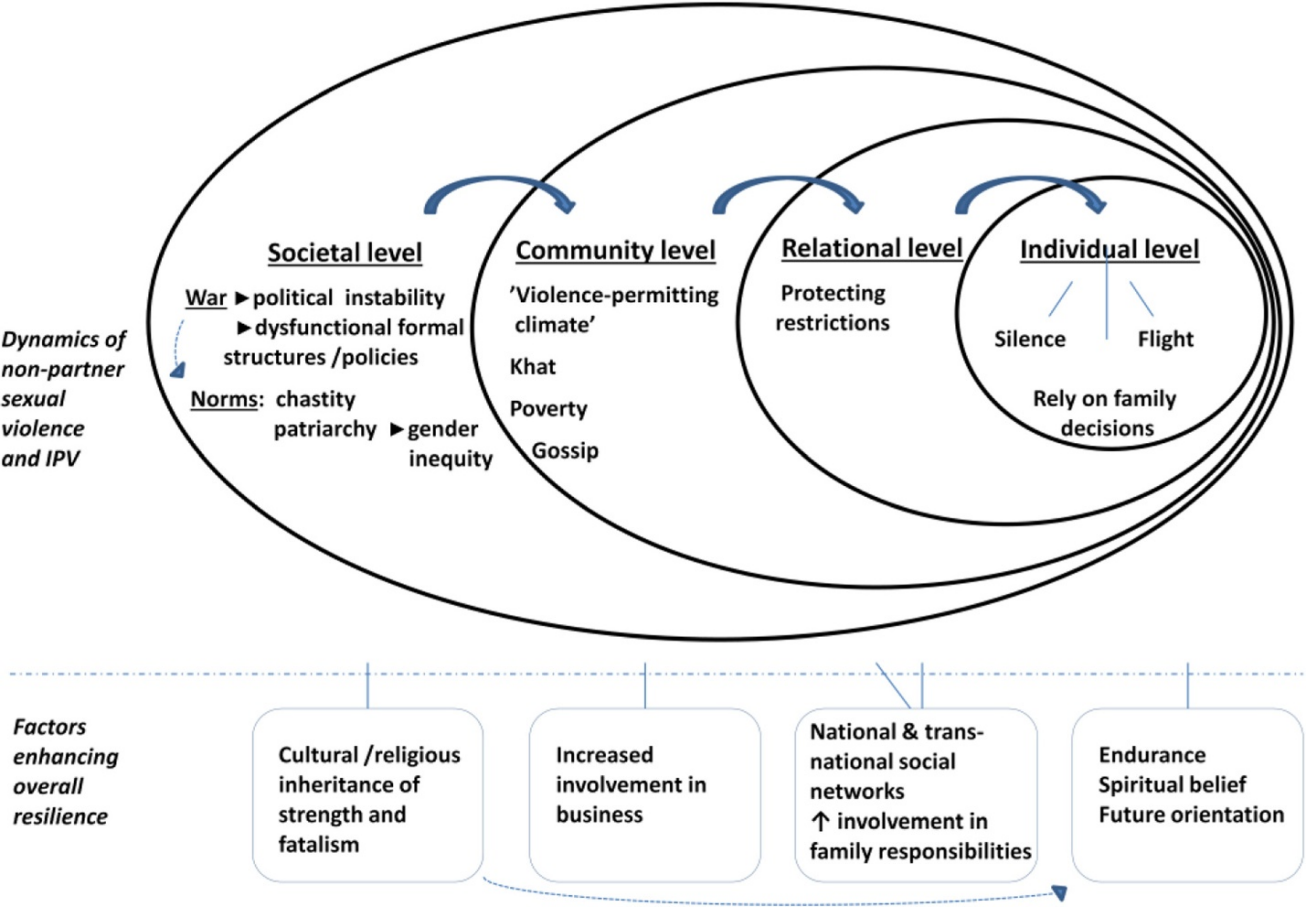
**Dynamics of non-partner sexual violence/IPV and women's overall resilience in the socio-ecological model (20).**

The integrated ecological framework [20, 21] may be used to illuminate the complexities of the silenced phenomenon of non-partner sexual violence and IPV, as shared by the informants. On a societal level, the lack of laws regulating violence perpetration and support for survivors have left ‘free rein’ to prevailing norms of chastity and gender inequity. The shame and stigma connected to non-partner sexual violence have their origin in these norms. Women’s chastity has traditionally been highly valued in the Somali society [29], and still prevails according to Somali women in diaspora [30]. Similar patterns of stigmatization connected to chastity are found in other settings of war rape [31, 32]. Informants linked a high risk of exposure to IPV among women to the societal patriarchal structure and unequal gender power relations. This link is confirmed by studies in other Sub-Saharan Africa countries [33, 34] and in Asia/the Pacific [35]. At a community level, the present study revealed how the war has contributed to a ‘violence-permitting’ climate, in addition to other risk factors such as khat use and poverty [36]. Another prominent finding on community level was the fear of gossip in community. Social risks of sharing experiences of war-driven sexual violence in Congo [31], Sudan [37] and Bosnia-Herzegovina [32] are described as subsequently reinforcing secrecy and avoidance of health care-seeking at both relationship and individual levels [37]. Our informants described how these social risks have created restrictions for women, motivated by protection rather than control. The families’ fear of ongoing warfare has further reinforced these restrictions. On an individual level, women exposed to non-partner sexual violence or IPV have been left with few choices: accept, remain silent, rely on family decisions, or flight (Figure 1).

### Capacity to cope with adversities

Silence after own exposure to sexual violence or IPV was prominent. However, women’s agency when handling the consequences of the overall violence in society and community was described and several factors enabling women’s coping, survival, and resilience were identified.

The concept of resilience has been used in a variety of settings to theorize individual capability coping with adversities in life [38, 39]. Payne [38] has argued that both psychological and physical sites for resilience emerge as a function of complex socioeconomic and political factors and there are individual and structural factors in the dynamic process of resilience [38]. In the present study, the lack of formal functional structures on societal level such as education, laws, and sufficient access to work and health care appeared to be compensated by strong national and transnational social networks at community and relationship levels. Part of the process of resilience was women’s increased involvement in business at a community level and overarching family responsibilities at a relationship level. This engagement increased during men’s war-related absence, and sharpened women’s strength; the same pattern was also found in Somaliland after the first decade of war [40].

Informants in the present study described how the longing for peace and life in a functioning society could result in “buufis”. Entering into the state of ‘buufis’ can be seen as an individual and temporary site of psychological resilience: a glimpse of a future elsewhere seen from an environment without prospects. This was sometimes proved successful if the goal of escape was achieved, but was devastating for general well-being if not. Ambiguous feelings connected to the ‘buufis’ phenomenon has previously been described among camp-based Somali’s in Kenya [41]. Individual conditions and skills become more crucial for enhancing resilience when structural conditions are limited or fail to provide support. Being a woman of strength was described as fundamental in the present study and the approaches of ‘moving on’ and endurance were highly valued strategies. A similar ‘moving on’ approach has been found among Somali born women in the United Kingdom [42] and is described as helpful in recovery after torture [43]. Endurance was connected to both personal inner capabilities and a trust in God whatever happens, thus, constituting acceptance and a resource of hope. The spiritual component in hope is documented among refugees from Sudan and Congo [44, 45] and is positively correlated to posttraumatic growth among Somali refugees in Hungary [46]. The factors enhancing resilience in the present study are presented in Figure 1.

The study revealed paradoxical roles of religion; a site of resilience for the individual but was a contributor to women’s difficulties in achieving agency when combined with power. The prominent finding of power-based restrictions, based on religious interpretations, undermine women’s access to sexual and reproductive health care services from an already insufficient level of health care provision within the country [47] and are a fundamental violation of basic human rights [48].

### Implications for health and health care in receiving countries

Somali refugee women’s pre-migration exposure to inadequate health care and silence concerning sexual violence/IPV can impact their health and attitudes towards health care in receiving countries [22]. In our study it appeared that informant’s experiences of preventive antenatal care was limited. This might imply an increased risk of existing but unknown health problems among newly arrived Somali-born women in Sweden. Furthermore, unfamiliarity with the Swedish health care system might contribute to the delayed antenatal care seeking found in a previous study [14]. The ‘move on’ approach and stoicism might further pose a barrier towards violence disclosure. Hence, it is a delicate balance for health care providers to on the one hand to draw upon endurance, social networks and future-orientation to strengthen newly arrived refugees, while on the other hand be open to individual needs related to a violent history.

A long-term adaption to a health care system dealing with only physical ill-health care was revealed in the present study. Mental illness is considered a ‘new’ problem encountering Somali communities after war [49]. Among Somali refugee women in Uganda [50] mental distress as a result of war is reported to continue years after violence exposure; however, Somali born informants in Sweden consider mental health services the “last choice” after traditional religious rituals and social support [51]. Thus, multiple factors contribute to silence and different health care seeking behavior in receiving countries. With respect to the link between violence, mental distress and adverse SRH [4, 17] further research is needed to identify suitable strategies for investigating and addressing Somali born women’s possible needs. The establishment of a relationship throughout motherhood between the antenatal care (ANC) midwife and the Somali-born woman is a potential site for mutual dialogue on health, violence exposure, and well-being.

### Methodological considerations and limitations

Throughout the study process, measures were taken to achieve trustworthiness [25]. Credibility was addressed through a varied selection of informants and the inclusion of both personal stories and vignette-based conversations, which allowed informants’ reflections from different perspectives. A thorough description of the informants’ characteristics and the choice of a well described structured analytic method [26] addressed the issue of transferability. To ensure consistency, the same investigators were retained throughout the study process and continuous field notes provided the possibility to check the reliability of the emergent design. In qualitative research, the researchers’ pre-understanding may influence findings and thus affect the confirmability [25]. One limitation of the present study might be the principal investigator being an outsider (a white Swedish midwife) which might have influenced both content and the amount of data gathered. However, this was partly balanced by the introduction of the vignette (Asma’s story) which served as an icebreaker to promote richer reflections from the informants’ sides on sensitive topics. Dialogue between the main investigator and the interpreter immediately after each interview addressed the influences of pre-understanding and served to achieve nuances. Furthermore, throughout the study process, all authors participated in several discussions, in which quotes verifying the probability of the interpretations were chosen. The language-barrier was another limitation of the study, but the inclusion of newly arrived informants and thus with limited Swedish-skills was important for achieving perspectives from more recent years in Somalia. The language interpreters were well respected within the Somali network, this may have rendered informants less willing to share sensitive or non-socially acceptable information. However, we consider the interpreters’ skills of creating a confident atmosphere were crucial during the interviews. Two independent interpreters were engaged to crosscheck and verify Somali translations.

## Conclusions

Several factors reinforced non-disclosure of violence exposure; therefore, after migration, violence-related illness might be overlooked in the health care system. Power-based restrictions reduced the possibilities for adequate health and care during pregnancy and childbirth. Social networks, increased engagement in public life, future-orientation, endurance, and faith were prominent sites for resilience during war. These features can contain resources for enhanced well-being and SRH in receiving countries after migration and need to be addressed in future interventions and research.
